# Reliability testing of a modified MISTELS score using a low-cost trainer box

**DOI:** 10.1186/s12909-019-1572-4

**Published:** 2019-05-06

**Authors:** Anis Hasnaoui, Haithem Zaafouri, Dhafer Haddad, Ahmed Bouhafa, Anis Ben Maamer

**Affiliations:** Department of General Surgery, Habib Thameur Hospital, Ali Ben Ayed, Street 2037 Montfleury, Tunis, Tunisia

**Keywords:** Training, Evaluation, Simulation, FLS, MISTELS, Reliability, Low-cost, Trainer box

## Abstract

**Background:**

Training programs such as the fundamentals of laparoscopic surgery (FLS) that are based on simulation are being currently used in several western countries. FLS allows skill acquisition and evaluation of competency in laparoscopic surgery. On the practical side, evaluation is determined by the MISTELS metrics (MISTELS is the acronym for the McGill inanimate system for training and evaluation of laparoscopic skills).

This training program may be modified so that it can be implemented in countries with limited resources using a low-cost trainer box. Would the use of a low-cost trainer box alter the reliability of the MISTELS score?

**Objective of study:**

The aim of the study was to evaluate the reliability of a modified MISTELS using a low-cost trainer box.

**Methods:**

It was a prospective study carried out at Habib Thameur hospital in Tunis (Tunisia), between April 2016 and August 2016. The study involved residents from different surgical specialties in the departments of general surgery and paediatric surgery of the hospital during 2015 and 2016.

This study assessed the reliability of a modified MISTELS system (Only three tasks were performed out of the five tasks used in the original MISTELS system). Evaluation was based on Cronbach’s alpha and intraclass correlation coefficients (ICC).

A low-cost trainer box was designed and constructed. The residents included in the study performed three series of three tasks using this trainer box. The first series was scored by two trained raters to evaluate inter-rater reliability. The two-other series were successively performed to evaluate test-retest reliability.

**Results:**

The internal consistency, assessed by Cronbach’s alpha, was at 0.929 which is an acceptable score. As for inter-rater and test-retest reliabilities that were assessed by ICCs, they yielded excellent scores that were at 1 and 0.95 (95% CI, 0.891–0.978) respectively.

**Conclusions:**

The reliability of a modified MISTELS is not altered by the use of a low-cost trainer box. The score of the modified MISTELS is a reliable score for evaluating technical skills of surgical residents using a low-cost trainer box.

**Electronic supplementary material:**

The online version of this article (10.1186/s12909-019-1572-4) contains supplementary material, which is available to authorized users.

## Background

In Tunisia, acquisition of technical skills in surgery is based on observation of senior surgeons in the operating room. As for assessment, it is based on evaluation of theoretical knowledge, leaving out the practical side.

In some western countries, simulation has become an essential part of the training program for young surgical residents in laparoscopic surgery. The fundamentals of laparoscopic surgery (FLS) is a program based on the McGill inanimate system for training and evaluation of laparoscopic skills (MISTELS) [1] which allows better acquisition of theoretical and practical skills in laparoscopic surgery as well as evaluation of these skills [2–4]. On the practical side, participants perform five tasks using a trainer box, to improve the basic skills of laparoscopic surgery. Once the tasks performed, a score based on evaluation of the time of execution and precision is given: the score of MISTELS.

This program may be modified using a low-cost trainer box and consumables, so that it can be used in countries with limited resources like ours. But would these modifications alter the reliability of the score of MISTELS.

The aim of this study was to assess the reliability of a modified MISTELS score, based on the performance of three tasks instead of five, using a low-cost trainer box, and to discuss the importance of low-cost simulators in surgical training.

## Methods

It was a prospective study carried out at Habib Thameur hospital in Tunis (Tunisia) between April 2016 and August 2016. The study included surgical residents of different surgical specialties in the departments of general surgery and paediatric surgery during the period 2015–2016. We excluded from the study the trainees who couldn’t participate or complete all the tasks.

Evaluation of the reliability was based on Cronbach’s alpha [5] which assessed the internal consistency of the scores assigned to different tasks in a single series, and the intraclass correlation coefficient (ICC) [6] which assessed the correlation between the score of different series.

**Fig. 1:**
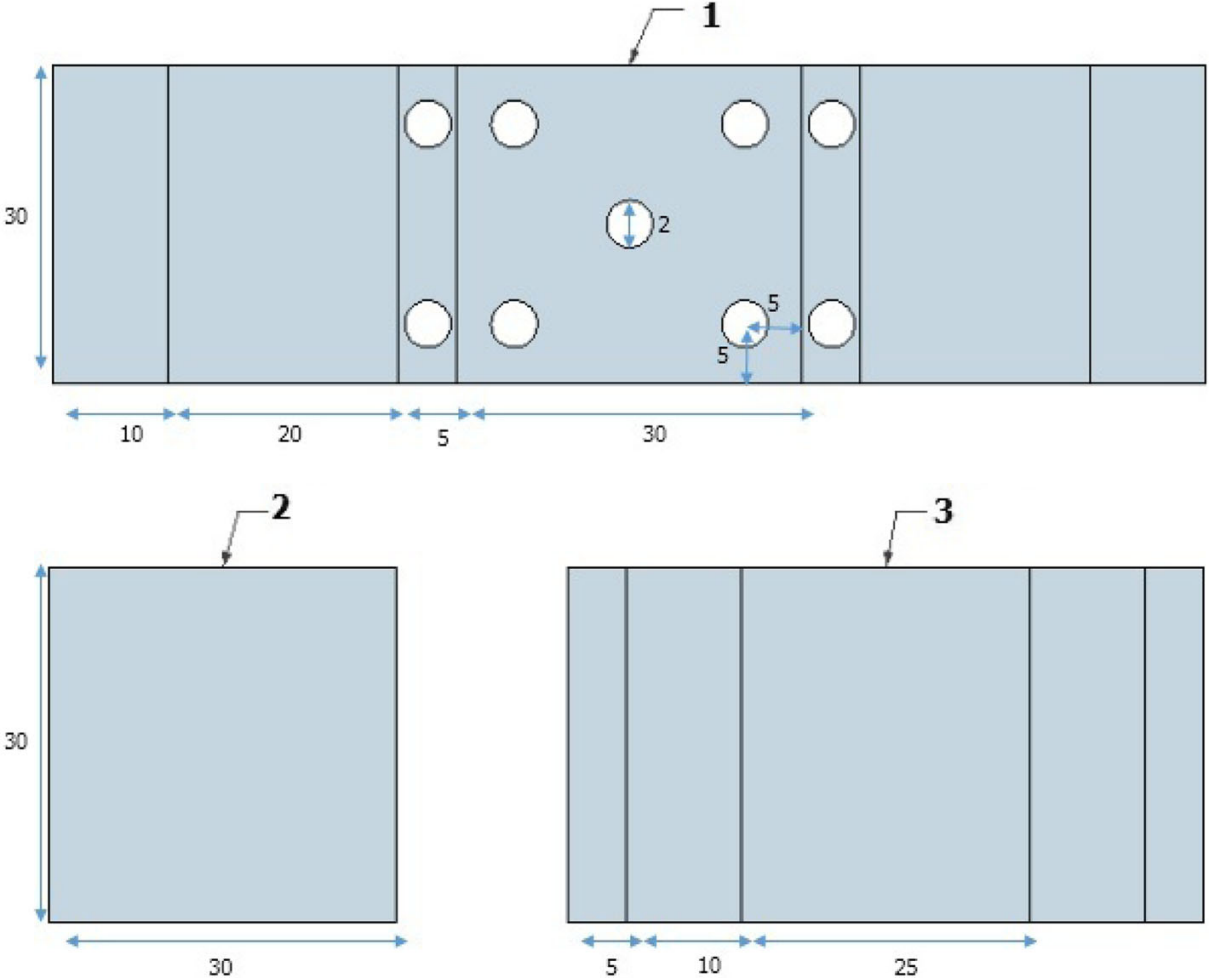
Measurements in cm of the plates and folding lines

**Fig. 2:**
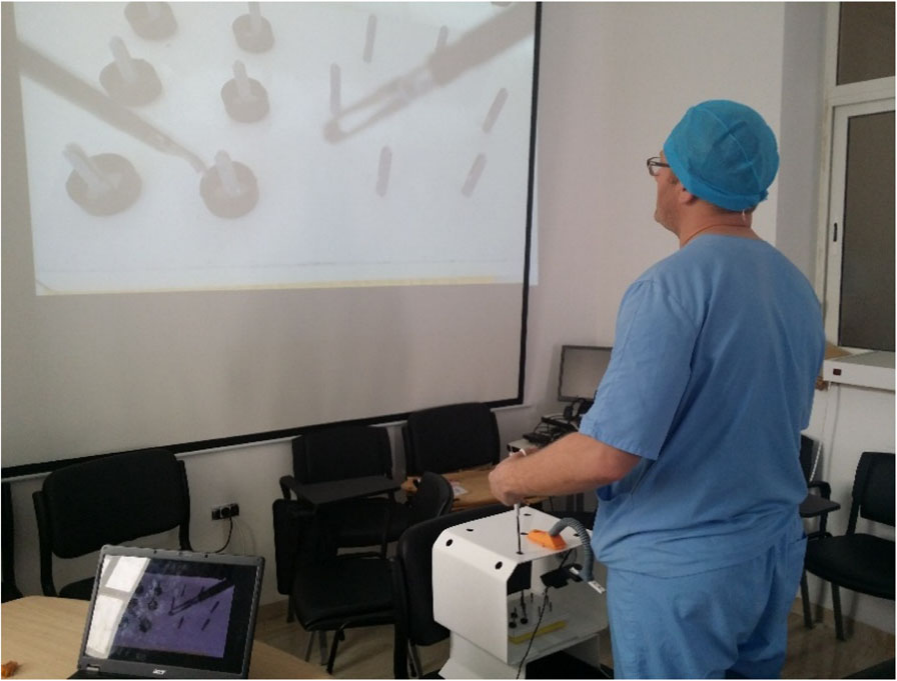
Images shown in real time onto the screen of the computer and the wall screen

Using a linear folder, we shaped rectangular plates of acrylic glass (Fig. 1) to obtain a trainer box measuring 30*37*23.5 cm (width*length*height). A high definition webcam (Logitech C920 HD Pro) was used in the study. To fix it on the trainer box, we used a holder of a desk lamp. Two LED lamps provided lighting. The cost of the box was estimated at 500 Tunisian Dinars (Which is the equivalent of $165 or £132.33 using the exchange rate on 06th January 2019), not counting the cost of the laparoscopic forceps. A portable computer was used to obtain the images from the webcam. The streaming of the images was obtained by the multimedia reader VLC 2.2.4 which is a freeware. Laparoscopic surgical instruments (Maryland forceps, a grasper, scissors and a needle holder) were used (Fig. 2). To videotape the performed tasks, we used the camera of a smartphone facing the central opening of the trainer box.

**Fig. 3:**
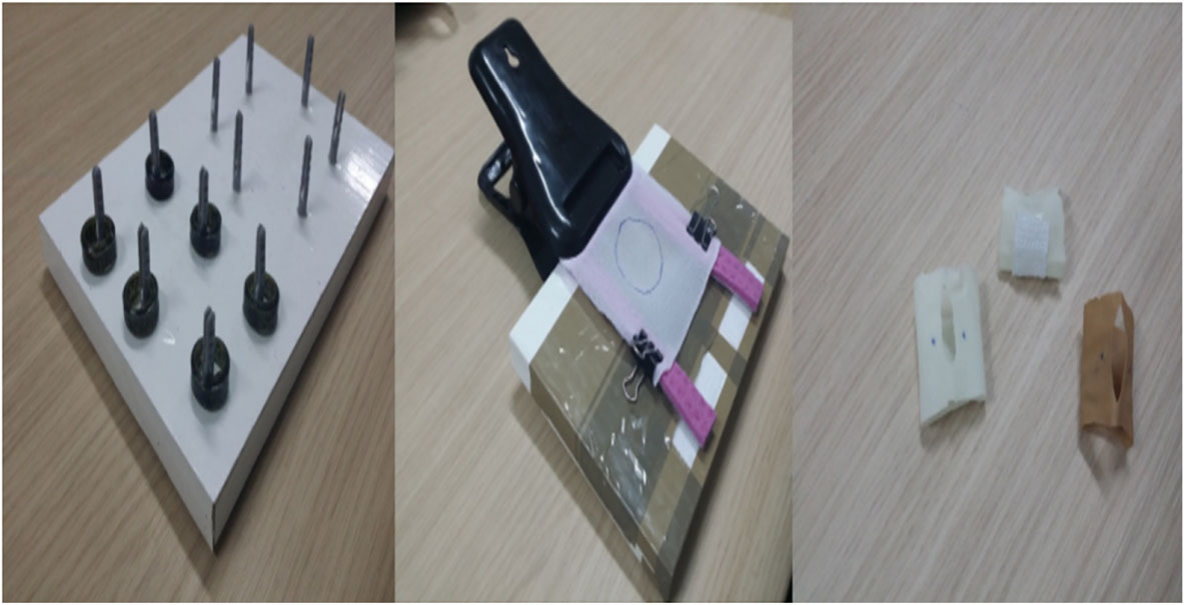
Materials used during the performance of the MISTELS tasks

The residents were scored on their performance of three tasks from the MISTELS program: peg transfer, precision cutting and suture with intra-corporeal knot (Fig. 3).

Each resident had a simulation session comprising 3 phases: briefing, simulation and debriefing.

During briefing, we explained to the residents the different aspects of the session: progress, technique and pointers. After the briefing, each resident performed three series of three tasks (peg transfer, precision cutting and suture with intra-corporeal knot) as stated in the literature [1]. The two other tasks of the MISTELS program (Ligating loop and suture with extra-corporeal knot) were not performed due to financial constraints. Debriefing aimed at underlining the positive and negative aspects of the session, laying down the acquired knowledge and bringing out the possible improvements that should be made.

Assessment of the performance of the residents was done in conformity with the data described in the literature [1]. The score for each task was normalized by dividing the score obtained by a predetermined standard value that was derived from the maximum score achieved by a chief resident for that task and then multiplying by 100.

For the first series of task, calculation of the scores was done by two raters to study the reliability between two raters. The first rater scored the performance on the spot at the end of each task. The second rater, who was blinded, scored the performances based on videotapes and tools kept intact after the tasks. The two-other series were successively performed and were scored by a single rater to study test-retest reliability. Thus, we obtained four scores for each resident (rater 1, rater 2, test and retest scores).

For statistical analysis, we used the SPSS version 21.0.0.0 software. Quantitative variables were expressed in averages. Internal consistency was determined using Cronbach’s alpha. Internal consistency was determined for the 4 series (rater 1, rater 2, test, retest) using the basic scores obtained for the three tasks (peg transfer, precision cutting and suture with intra-corporeal knot) in each series. The deletion impact of each of the three tasks on the internal consistency was evaluated in the last series (retest). Test-retest and inter-rater reliabilities were determined by the ICC. Each ICC was calculated using a two-way mixed model with absolute agreement and a 95% confidence interval.

## Results

All the results of the study are presented in Additional file 1. The study involved 26 residents having different surgical specialties. One of the residents was excluded from the study because he didn’t perform all the tasks. Distribution of residents by specialty was as follows: fourteen residents of general surgery, five of paediatric surgery, two of urology, two surgical residents requesting for an equivalent rating of their diploma, a resident of surgical oncology and a resident of gynaecology. The residents were aged 30.08 years on average (range 27–38). Nineteen of the residents had already had some experience in laparoscopic surgery.

To assess the internal consistency of the MISTELS score, we calculated Cronbach’s alpha coefficient for each of the series (rater 1, rater 2, test, retest). The obtained Cronbach’s alpha coefficients were all acceptable. Table 1 shows the correlation of each task with the total score achieved in the last series (retest), as well as the deletion impact of each task on Cronbach’s alpha coefficient.

**Table 1:** Correlation between the score of each task and the total score and impact of deletion of each task on Cronbach’s alpha and thus on internal consistency (Scores obtained in the series of retest)

Tasks	Correlation with the total score	Cronbach’s alpha in case of deletion of the exercise
Peg transfer	0.972	0.832
Precision cutting	0.894	0.951
intra-corporeal knot	0.952	0.889

Scores of each task had a high correlation with the total score. Deletion of the second exercise (precision cutting) would slightly improve Cronbach’s alpha (0.951). Deletion of the two other tasks would, on the other hand, lower it.

Inter-rater reliability was evaluated using the ICC. This coefficient was equal to one which confirmed the low variability between the scores of two raters (Table 2).

**Table 2:** Internal consistency assessed by Cronbach’s alpha and inter-rater reliability assessed by ICC of the modified MISTELS score

Tasks	Rater 1		Rater 2		ICC^b^
	Mean	SD^a^	Mean	SD	
Peg transfer	32.49	30.22	32.38	30.12	1
Precision cutting	20.80	24.61	20.63	24.62	1
intra-corporeal knot	27.42	29.58	27.5	29.70	1
Total score	80.72	81.14	80.52	81.02	1
Cronbach’s alpha	0.955	0.953			

^a^*SD* Standard deviation, ^b^*ICC* Intraclass correlation coefficient

**Fig. 4:**
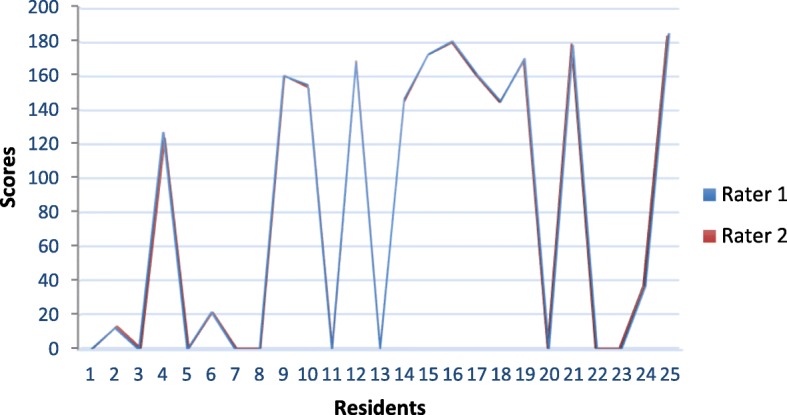
Modified MISTELS scores given to residents by two raters

The total scores granted by each rater are represented by curves in Fig. 4. Both curves are matching which confirms the low variability between raters.

The test-retest reliability was excellent with an ICC of 0.95 (95% CI, 0.891–0.978) (Table 3).

**Table 3:** Internal consistency assessed by Cronbach’s alpha and test-retest reliability assessed by ICC of the modified MISTELS score

Tasks	Test		Retest		ICC^b^
	Mean	SD^a^	Mean	SD	
Peg transfer	33.55	28.72	36.26	29.23	0.952
Precision cutting	26.69	24.09	32.96	23.07	0.769
intra-corporeal knot	31.45	32.47	28.96	31.17	0.902
Total score	91.69	80.65	98.18	78.70	0.950
Cronbach’s alpha	0.933		0.929		

^a^*SD* Standard deviation, ^b^*ICC* Intraclass correlation coefficient

**Fig. 5:**
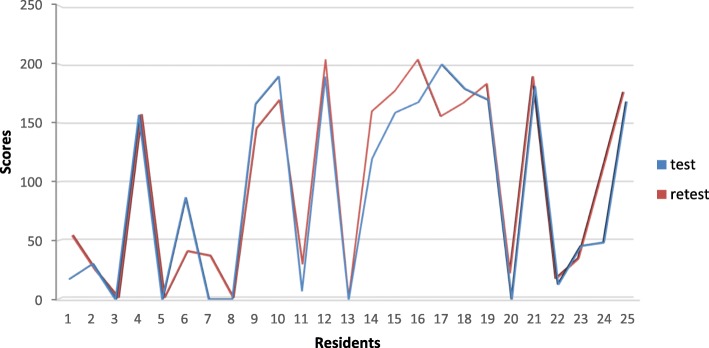
Modified MISTELS scores given to residents in the test and the retest series by a single rater

The scores granted in the test and retest are shown in Fig. 5. The curves are not totally matching which indicates a difference, minimal as it may seem, in the scores obtained in the test and retest series.

## Discussion

### Reliability testing using a low-cost trainer box

Using a low-cost trainer box, we found that the modified MISTELS score is a reliable score for assessing competency in laparoscopic surgery.

Reliability refers to the consistency, stability and precision of a test results [7]. It is important to evaluate the reliability for two main reasons. Firstly, the necessity to establish evidence for reliability is driven by errors that characterize all instruments of measurement. The impact of these errors on the scores of a test is unpredictable for these errors occur at random. Using an unreliable test is like giving useless random scores to the participants in this test. Secondly, assessment of reliability always precedes the study of validity. In fact, an effective test should be reliable and valid [8]. Proving the validity of a test is a long process which requires several steps and experiments. Before embarking on such a procedure, we should first make sure that the measures are reliable. Otherwise, the results will be false.

The study of reliability comprises an assessment of the internal consistency based on the calculation of Cronbach’s alpha and assessment of the inter-rater and test-retest reliabilities by calculating the ICC.

#### Internal consistency

The internal consistency of a test ensures that all the components of a score are measuring the same thing [9]. It is considered satisfactory if alpha varies between 0.7 and 1 [9]. In this study, Cronbach’s alphas in the different series were superior to 0.9 which means that they were acceptable [9]. This suggests that the three performed tasks in our study measured repeatedly and constantly the resident’s competency in laparoscopic surgery and supports the use of the total score as a one-dimensional scale. By reconsidering the formula of this coefficient, we remark that the internal consistency is proportional to the length of the test [10]. However, in our study, we used three tasks out of the five tasks in the original MISTELS score. Spearman-Brown prophecy formula [11, 12] statistically predicts reliability of a modified test insofar as the added or subtracted items are qualitatively equivalent to those comprised in the initial test [13]. Based on this formula, Cronbach’s alpha coefficients would have better values than those found in our study.

In the literature, we found only one study that assessed the reliability of the MISTELS score [14]. It was carried out by Vassiliou et al. In their study involving 12 subjects, the best value of Cronbach’s alpha was achieved during the test. It was equal to 0.86, which is an acceptable value [9]. Cronbach’s alpha coefficients in our study were slightly superior to those reported in the literature. This may be explained by the scores obtained by the inexperienced residents in our study, those who obtained a score of zero in all the exercises of a given series. The number of these residents varied between 2 (In the series of retest) and 10 (In the series of scores given by both raters). If we delete the results of the two residents who obtained a score of zero in all the retest tasks, the Cronbach’s alpha coefficient in the series of retest would be 0.899. This is an acceptable value which is in accordance with the value reported in the literature.

#### Inter-rater and test-retest reliabilities

In the evaluation of the inter-rater reliability, the errors of measurement may be due to the different raters using different methods. In our study, we didn’t expect a significant difference between the scores given by the two raters for a same test, for measurements of time and precision were standardized. ICC used to assess inter-rater reliability was equal to one, confirming the low variability between raters. In the study of Vassiliou et al., the ICC for inter-rater reliability was at 0.998 (95% CI, 0.985–1). This result was in accordance with our result.

As for the assessment of test-retest reliability, errors of measurement can be grouped into three categories: Errors associated with the raters, errors associated with the equipment used for the test and errors associated with the residents themselves (Different performances due to the effect of practice or to fatigue, distraction and frustration). Errors associated with the raters and with the used equipment were limited by standardizing methods of measurement by the raters and using predefined measurements for the equipment used in the tasks. As for the performance of each resident, it may improve especially for beginners (MISTELS is a training program that is supposed to improve competency in laparoscopic surgery [1, 15, 16]). Or it may deteriorate due to fatigue or frustration (Hence the importance of motivating the residents). To minimize these errors, the test and retest were performed successively without free interval. Nevertheless, two novice residents had a score of zero in the test but improved their score in the retest. ICC of the test-retest was excellent with a value of 0.95 (95% CI, 0.891–0.978). In the study carried out by Vassiliou et al., the ICC of the test-retest was slightly inferior to the value reported in our study. It was equal to 0.892 (95% CI, 0.665–0.968) [14]. According to Vassiliou et al., ICC of the test-retest was inferior to the ICC of the inter-rater reliability due to the effect of practice on the performance of the trainees. In fact, it was found that practice using MISTELS trainer improved the score of trainees even after a single training session [1, 15, 16]. This improvement was more marked in the less experienced residents. To decrease the effect of practice on performance, Vassiliou included the residents who had achieved a total score (including the five tasks of MISTELS) of 230. In addition, the test and retest series were performed successively. In our study, the effect of practice on performance was not so well marked. The ICC of the test-retest in our study was higher than the ICC reported in Vassiliou’s study; which raises the question of the effectiveness of our model in comparison to the original model of MISTELS. The answer to this question would probably require further research study to test the validity of our model.

### The importance of low-cost simulators in surgical training

Simulation has become the corner stone of surgical training in many developed countries. Many scores have been introduced using different models ranging from low fidelity and low-cost trainers to high fidelity simulation trainers and animal models. This wide range raised the debate of the most effective and cost/effective model.

Many studies proved the effectiveness of low-cost trainers in surgical training [17] and their reliability in the evaluation of training [18]. Chandrasekera et al. used a randomised blinded study to compare the effectiveness of a cheap training model using a cardboard box and a conventional video-laparoscopy trainer [19]. The authors found no significant difference between the two models. In their study, the cost of the conventional trainer was 30.000 euros. Thus, they concluded that the cardboard box is more efficient (Efficiency as the relationship between cost and effectiveness) in the training. Sandberg et al. used a randomised controlled trial to validate a non-anatomic and low-cost model for arthroscopy training [20]. This study concluded that the model is an effective knee arthroscopy trainer that may decrease the learning curve without significant cost.

It is obvious that the cost is the main advantage in using low-cost trainers. It offers novice surgeons the possibility of more regular training, even at home. In a systematic review of low-cost laparoscopic simulators, Mimi M. Li et al. identified 73 unique simulators (60 non-commercial, 13 commercial) [21]. The cost ranged from £3 to £216 for non-commercial and £60 to £1007 for commercial simulators. Forty-five per cent (27) of non-commercial simulators in this review were not subject to any validation test [21]. The cost of our model (Hardware and labor) was £132.33 using the exchange rate on 06th January 2019. We should mention that the cost of the webcam was around £85. Furthermore, the use of low-cost consumables, like in our study, could drastically reduce the cost of training. In a cost comparison study between standard FLS equipment and low-cost equipment, Franklin BR et al. found that the use of low-cost equipment results in significant financial savings and that a five-resident program will save approximately $8500 annually [22].

Thus, low-cost simulators are more efficient than high fidelity simulators, offering equivalent training results with lower cost. On the other hand, high fidelity simulators and animal models offer more realistic situations and variability for the trainees to deal with. Therefore, we encourage the conjunction of the different simulators for a complete training when it is possible.

## Conclusions

Even though additional evidence is still required, the value of simulation in laparoscopic surgery can by no means be ignored or underestimated [23]. Simulation should be offered to every resident as part of their training program. The score of the modified MISTELS is a reliable score for evaluating the technical skills of surgical residents using the trainer box that we have constructed. Keeping in mind the financial constraints in countries with limited resources, we consider that implementing FLS using low-cost materials can provide a solution to this problem that hinders the training of young residents.

## Additional file

Additional file 1: Results of the study containing the raw data used in the study. (XLSX 11 kb)

## Abbreviations

FLS: Fundamentals of laparoscopic surgery
ICC: Intraclass correlation coefficient
MISTELS: McGill inanimate system for training and evaluation of laparoscopic skills
